# Effectiveness and safety of direct oral anticoagulants compared to warfarin in treatment naïve non-valvular atrial fibrillation patients in the US Department of defense population

**DOI:** 10.1186/s12872-019-1116-1

**Published:** 2019-06-13

**Authors:** Kiran Gupta, Jeffrey Trocio, Allison Keshishian, Qisu Zhang, Oluwaseyi Dina, Jack Mardekian, Anagha Nadkarni, Thomas C Shank

**Affiliations:** 1 Bristol-Myers Squibb, Lawrenceville, NJ USA; 2 Pfizer Inc., New York, NY USA; 3 STATinMED Research, 211 N 4th Ave, Ste 2B, Ann Arbor, MI 48104 USA

**Keywords:** Non-valvular atrial fibrillation, Stroke/systemic embolism, Major bleeding, Warfarin, Direct oral anticoagulants

## Abstract

**Background:**

Clinical trials have demonstrated that direct oral anticoagulants (DOACs) are at least non-inferior to warfarin in reducing the risk of stroke/systemic embolism (SE) among patients with non-valvular atrial fibrillation (NVAF), but the comparative risk of major bleeding varies between DOACs and warfarin. Using US Department of Defense (DOD) data, this study compared the risk of stroke/SE and major bleeding for DOACs relative to warfarin.

**Methods:**

Adult patients with ≥1 pharmacy claim for apixaban, dabigatran, rivaroxaban, or warfarin from 01 Jan 2013–30 Sep 2015 were selected. Patients were required to have ≥1 medical claim for atrial fibrillation during the 12-month baseline period. Patients with a warfarin or DOAC claim during the 12-month baseline period were excluded. Each DOAC cohort was matched to the warfarin cohort using propensity score matching (PSM). Cox proportional hazards models were conducted to evaluate the risk of stroke/SE and major bleeding of each DOAC vs warfarin.

**Results:**

Of 41,001 identified patients, there were 3691 dabigatran-warfarin, 8226 rivaroxaban-warfarin, and 7607 apixaban-warfarin matched patient pairs. Apixaban was the only DOAC found to be associated with a significantly lower risk of stroke/SE (hazard ratio [HR]: 0.55; 95% confidence interval [CI]: 0.39, 0.77; *p* < 0.001) and major bleeding (HR: 0.65; 95% CI: 0.53, 0.80; p < 0.001) compared to warfarin. Dabigatran and rivaroxaban initiation were associated with similar risk of stroke/SE (dabigatran: HR: 0.68; 95% CI: 0.43, 1.07; *p* = 0.096; rivaroxaban: HR: 0.83; 95% CI: 0.64, 1.09; *p* = 0.187) and major bleeding (dabigatran: HR: 1.05; 95% CI: 0.79, 1.40; *p* = 0.730; rivaroxaban: HR: 1.07; 95% CI: 0.91, 1.27; *p* = 0.423) compared to warfarin.

**Conclusion:**

Among NVAF patients in the US DOD population, apixaban was associated with significantly lower risk of stroke/SE and major bleeding compared to warfarin. Dabigatran and rivaroxaban were associated with similar risk of stroke/SE and major bleeding compared to warfarin.

**Electronic supplementary material:**

The online version of this article (10.1186/s12872-019-1116-1) contains supplementary material, which is available to authorized users.

## Background

Atrial fibrillation (AF) is an independent risk factor for stroke and increased mortality [1]. It was estimated that 5.2 million US adults were affected by AF in 2010, while in 2015 the prevalence of AF was close to 9.6 million. This number is projected to increase to 12.1 million by 2030, corresponding to a growth rate of 4.3% [2, 3].

Warfarin, a vitamin K antagonist (VKA), has been the standard treatment for decades for stroke prevention among AF patients [4]. The American College of Cardiology/American Heart Association/ Heart Rhythm Society Guideline recommends oral anticoagulants (OACs) be used in patients with non-valvular AF (NVAF) and prior stroke, transient ischemic attack (TIA), or a CHA_2_DS_2_-VASc (congestive heart failure, hypertension, aged > 75 years, diabetes, prior stroke or transient ischemic attack, thromboembolism, vascular disease, aged 65–74 years, and gender) score ≥ 2 [5]. Besides warfarin, four direct oral anticoagulants (DOACs; dabigatran, rivaroxaban, apixaban, edoxaban) have received US Food and Drug Administration (FDA) approval. When compared with warfarin, DOACs have advantages of more predictable pharmacological profiles, fewer drug-drug interactions, an absence of major dietary effects, no requirement for regular international normalized ratio (INR) monitoring, and less risk of intracranial bleeding [5].

Four large prospective non-inferiority clinical trials have compared the effectiveness and safety between DOACs and warfarin among NVAF patients [6–9]. In the RE-LY trial, those prescribed 150 mg dabigatran had lower rates of stroke/SE and similar rates of major bleeding compared to warfarin [6]. The ROCKET AF trial showed that patients prescribed rivaroxaban had non-inferior rates of stroke/SE and similar rates of major bleeding [7]. In the ARISTOTLE trial, apixaban demonstrated superiority to warfarin with lower rates of stroke/SE and major bleeding [8]. The ENGAGE AF trial showed that patients prescribed edoxaban had non-inferior rates of stroke/SE and lower rates of major bleeding compared to warfarin [9].

In addition to clinical trials, several real-world studies have evaluated comparative effectiveness and safety between DOACs and warfarin [10, 11]. Being one of the largest health care plans in the US, the analysis of the US Department of Defense (DOD) health care system adds evidence and complements the profile in understanding the real-world treatment effects of OACs among NVAF patients in the US. However, few real-world studies using the DOD data have been conducted between DOACs and warfarin. The aim of this study was to compare the risk of stroke/SE and major bleeding between DOACs and warfarin in the DOD data.

## Methods

### Data source

This retrospective observational study used the US DOD data from January 1, 2012 to September 30, 2015. The DOD provides health care to over 9.4 million beneficiaries located in all 50 US states and multiple countries globally. Eligible beneficiaries include active duty, activated guard and reserve, retirees, survivors, some inactive guard and reserve, and their family members. Most beneficiaries are retired service members and their family members (5.42 million, 57%), many of whom are Medicare eligible (3.18 million). Beneficiaries remain in the system for an average length of 7.2 years, which is 2–3 times longer than commercial insurance plans. The data repository includes comprehensive datasets providing integrated information about the inpatient, outpatient, ER, and pharmacy claims from the US DOD facility and civilian/private sector care for eligible beneficiaries.

Medical and pharmacy claim coding utilizes the National Drug Code (NDC) coding system, Healthcare Common Procedure Coding System (HCPCS) codes, Current Procedural Terminology (CPT) codes, and the International Classification of Disease, 9th Revision, Clinical Modification (ICD-9-CM).

### Study population

This study selected adult patients with ≥1 pharmacy claim for an OAC (warfarin, apixaban, dabigatran, or rivaroxaban) from January 1, 2013 to September 30, 2015 (identification period). Edoxaban was not included in the analysis due to small sample size (*N* = 131). The first DOAC prescription claim date was defined as the index date for patients with a DOAC claim(s). For those without a DOAC claim, the first warfarin prescription claim date was defined as the index date. The baseline period was defined as one-year before the index date, during which patients had ≥1 medical claim for AF (ICD-9-CM: 427.31) and continuous enrolment [12]. Patients were excluded from the study if they had claims for valvular heart disease, heart valve replacement, dialysis, kidney transplant, end-stage chronic kidney disease, venous thromboembolism, reversible AF, or a pharmacy claim for an OAC during the baseline period, hip or knee replacement within 6 weeks prior to the index date, > 1 OAC claim on the index date, or a pregnancy diagnosis during the study period (Additional file 1: Table S1).

The follow-up period was defined as one day after the index date until the earliest of the following dates: OAC discontinuation date (≥30-day gap between OAC prescriptions), switch to a non-index OAC < 30 days before or after discontinuation, death, end of continuous medical and pharmacy enrollment, or end of study period [13].

### Outcome measures

Defined by primary or secondary diagnosis position on inpatient claims, stroke/SE was utilized as the effectiveness outcome measure while major bleeding served as the measure for safety outcomes. Stroke/SE was further classified into ischemic stroke, hemorrhagic stroke, and SE. Major bleeding consisted of intracranial hemorrhage (ICH), gastrointestinal (GI) bleeding, and major bleeding at other key sites. Validated administrative claim-based algorithms as well as published articles were used to derive the stroke/SE and major bleeding code lists. (Additional file 1: Table S2) [14–17].

### Baseline variables

Baseline measurements included patient demographics, comorbidities, medications, hospitalizations during the 12-month baseline period, and clinical risk scores (HAS-BLED [hypertension, abnormal kidney or liver function, stroke, bleeding, age > 65 years, and drugs/alcohol abuse or dependence], Charlson Comorbidity Index [CCI], and CHA_2_DS_2_-VASc).

The CHA_2_DS_2_-VASc stroke risk score and HAS-BLED bleeding risk scores were calculated (Additional file 1: Table S3 and Table S4) [18, 19]. Note that for the HAS-BLED score, INR and other lab values were unavailable in the data; a modified score (range 0 to 8) was used.

### Statistical methods

The design, analytical methods, and presentation of this study were informed by the guidelines for comparative effectiveness research [20, 21].

To assess significant differences for dichotomous variables, Pearson’s Chi-square tests were performed. For continuous variables, student t-tests were used.

To control for potential confounders between comparative cohorts (apixaban vs warfarin, rivaroxaban vs warfarin, and dabigatran vs warfarin), one-to-one propensity score matching (PSM) was used to balance demographics and clinical characteristics and to estimate the average treatment effects in patients with similar characteristics for whom each of the two OACs would be a reasonable treatment choice [22]. The logistic regression for the propensity score calculation included inpatient admissions, baseline medication use, age, gender, US geographic region, CCI score, HAS-BLED score, CHA_2_DS_2_-VASc score, stroke and bleeding history, and comorbidities [23]. The nearest neighbor method without replacement with a caliper of 0.01 was used. The balance of baseline patient characteristics was checked based on mean standardized differences with a threshold of 10% [24].

Incidence rates per 100 person-years of stroke/SE and major bleeding in PSM matched cohorts were calculated. To assess the risk of stroke/SE and major bleeding for patients in the matched cohorts, Cox proportional hazards models were utilized. Hazard ratios, 95% confidence intervals (CIs), and *p*-values were provided. OAC treatment was included as the independent variable, and no other covariates were included in the model because the cohorts were balanced.

### Sensitivity analyses

Sensitivity analyses, for the purpose of testing the robustness of the main results, were conducted. In the first of these analyses, cohorts were stratified by dosage of DOACs (standard and reduced) on the index date to assess if the outcomes were altered by DOACs dosage. The post-PSM population was separated per dosage of DOACs on the index date: standard-dose (apixaban 5 mg-warfarin, rivaroxaban 20 mg-warfarin, and dabigatran 150 mg-warfarin) and reduced-dose (apixaban 2.5 mg-warfarin, rivaroxaban 15 mg-warfarin, and dabigatran 75 mg-warfarin). In each matched subgroup by dosage of DOACs, imbalanced baseline variables with standardized difference > 10% were included in the Cox proportional hazards models. The statistical significance of the interaction term between treatment and dose was determined with a cutoff point of *p*-value = 0.10.

Second, patients who had catheter ablation within 2 months prior to the index prescription and those who had cardioversion 1 month before or after index drug were excluded. After excluding those patients, the balance of the baseline characteristics was checked and variables which were unbalanced were incorporated in the multivariate model. These patients were excluded because they likely had a low risk of stroke and received the OACs for the procedures and not long-term stroke prevention. Third, a sensitivity analysis using the 6-months after the index date as follow-up was also conducted. In this analysis, patients were censored at the earliest of: the OAC prescription discontinuation date, date of switching, date of death, date of disenrollment, end of the study period (September 30, 2015), or 6 months after the index date. This sensitivity analysis allowed the follow-up period to be more balanced between the cohorts. Lastly, a sensitivity analysis using the intent-to-treat method was used, where patients were followed based on the index drug regardless of discontinuation or switch.

## Results

### Baseline characteristics

**Fig. 1:**
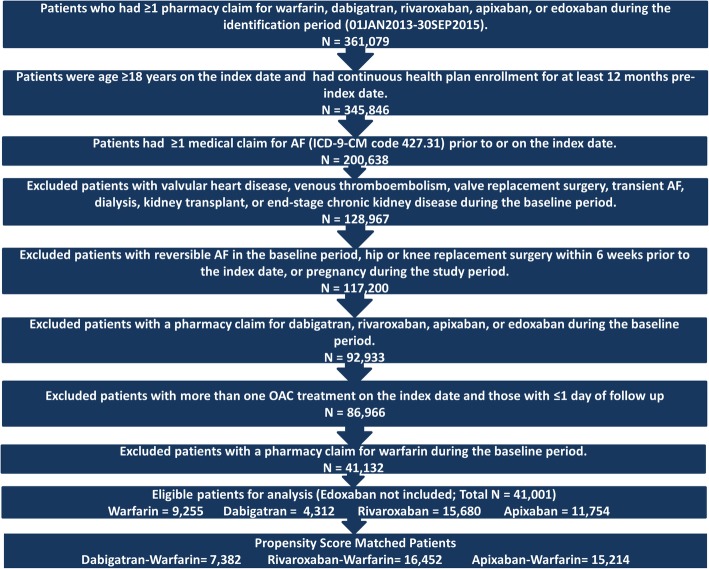
Patient Selection Figure. AF: atrial fibrillation. OAC: oral anticoagulant

After applying the selection criteria and before performing the PSM, a total of 41,001 patients were included in the study, including 9255 (22.6%) warfarin, 4312 (10.5%) dabigatran, 15,680 (38.2%) rivaroxaban, and 11,754 (28.7%) apixaban patients. Warfarin initiators were older with significantly higher baseline mean CCI and CHA_2_DS_2_-VASc scores vs those who initiated apixaban, rivaroxaban, and dabigatran. After 1:1 PSM, there were 3691 dabigatran-warfarin matched pairs, 8226 rivaroxaban-warfarin matched pairs, and 7607 apixaban-warfarin matched pairs (Fig. 1).

After PSM, baseline demographic and clinical characteristics were balanced between the matched cohorts with standardized difference less than 10%. Dabigatran-warfarin patients had the best health status with a mean CCI score of 2.0, CHA_2_DS_2_-VASc score of 3.7, and HAS-BLED score of 2.8, followed by rivaroxaban-warfarin and apixaban-warfarin patients with a mean CCI score approximately 2.5, CHA_2_DS_2_-VASc score around 4.1, and HAS-BLED score of 3.0 (Table 1).

**Table 1:** Demographic and Clinical Characteristics in Propensity Score Matched DOAC and Warfarin Cohorts

	Warfarin Cohort (*N* = 3691)		Dabigatran Cohort (*N* = 3691)		Warfarin Cohort (*N* = 8226)		Rivaroxaban Cohort (*N* = 8226)		Warfarin Cohort (*N* = 7607)		Apixaban Cohort (*N* = 7607)	
	N/Mean	%/SD	N/Mean	%/SD	N/Mean	%/SD	N/Mean	%/SD	N/Mean	%/SD	N/Mean	%/SD
Age	74.0	10.3	74.0	9.5	76.5	9.7	76.5	9.3	76.6	9.8	76.5	9.5
18–54	134	3.6%	106	2.9%	180	2.2%	168	2.0%	178	2.3%	172	2.3%
55–64	425	11.5%	434	11.8%	625	7.6%	590	7.2%	563	7.4%	558	7.3%
65–74	1244	33.7%	1286	34.8%	2278	27.7%	2275	27.7%	2057	27.0%	2157	28.4%
≥ 75	1888	51.2%	1865	50.5%	5143	62.5%	5193	63.1%	4809	63.2%	4720	62.0%
Gender												
Male	2240	60.7%	2245	60.8%	4812	58.5%	4791	58.2%	4430	58.2%	4431	58.2%
Female	1451	39.3%	1446	39.2%	3414	41.5%	3435	41.8%	3177	41.8%	3176	41.8%
Geographic Region												
Northeast	270	7.3%	282	7.6%	807	9.8%	788	9.6%	644	8.5%	632	8.3%
North Central	559	15.1%	566	15.3%	1409	17.1%	1377	16.7%	1191	15.7%	1182	15.5%
South	1862	50.4%	1865	50.5%	3701	45.0%	3709	45.1%	3688	48.5%	3672	48.3%
West	929	25.2%	911	24.7%	2138	26.0%	2174	26.4%	1933	25.4%	1966	25.8%
Other	71	1.9%	67	1.8%	171	2.1%	178	2.2%	151	2.0%	155	2.0%
Baseline Comorbidity												
Deyo-Charlson Comorbidity Index	2.0	2.1	2.0	2.1	2.5	2.4	2.5	2.4	2.5	2.4	2.5	2.4
CHADS2 Score	2.3	1.3	2.3	1.4	2.5	1.4	2.6	1.4	2.6	1.4	2.6	1.4
0 = low risk	309	8.4%	298	8.1%	453	5.5%	491	6.0%	408	5.4%	422	5.5%
1 = moderate risk	782	21.2%	835	22.6%	1408	17.1%	1459	17.7%	1294	17.0%	1318	17.3%
2 = high risk	1209	32.8%	1166	31.6%	2528	30.7%	2351	28.6%	2360	31.0%	2247	29.5%
>2 = high risk	1391	37.7%	1392	37.7%	3837	46.6%	3925	47.7%	3545	46.6%	3620	47.6%
CHADS2-VASc Score	3.7	1.8	3.7	1.8	4.1	1.7	4.1	1.8	4.1	1.8	4.2	1.8
0 = low risk	84	2.3%	99	2.7%	117	1.4%	117	1.4%	111	1.5%	108	1.4%
1 = moderate risk	303	8.2%	251	6.8%	414	5.0%	389	4.7%	377	5.0%	363	4.8%
2 = high risk	517	14.0%	562	15.2%	901	11.0%	931	11.3%	832	10.9%	872	11.5%
>2 = high risk	2787	75.5%	2779	75.3%	6794	82.6%	6789	82.5%	6287	82.6%	6264	82.3%
HAS-BLED Score	2.8	1.3	2.8	1.2	3.0	1.3	3.0	1.3	3.0	1.3	3.0	1.3
0 = low risk	92	2.49%	80	2.17%	117	1.42%	101	1.23%	110	1.45%	100	1.31%
1–2 = moderate risk	1544	41.8%	1472	39.9%	3007	36.6%	2982	36.3%	2737	36.0%	2606	34.3%
>2 = high risk	2055	55.7%	2139	58.0%	5102	62.0%	5143	62.5%	4760	62.6%	4901	64.4%
Baseline Prior Bleed	573	15.5%	572	15.5%	1611	19.6%	1632	19.8%	1484	19.5%	1525	20.0%
Baseline Prior Stroke	354	9.6%	341	9.2%	1034	12.6%	1028	12.5%	907	11.9%	931	12.2%
Congestive Heart Failure	749	20.3%	752	20.4%	2184	26.5%	2207	26.8%	2033	26.7%	2052	27.0%
Diabetes	1211	32.8%	1220	33.1%	2853	34.7%	2815	34.2%	2593	34.1%	2631	34.6%
Hypertension	3055	82.8%	3059	82.9%	6903	83.9%	6881	83.6%	6450	84.8%	6469	85.0%
Renal Disease	625	16.9%	640	17.3%	1918	23.3%	1943	23.6%	1839	24.2%	1852	24.3%
Myocardial Infarction	202	5.5%	204	5.5%	513	6.2%	525	6.4%	479	6.3%	485	6.4%
Dyspepsia or Stomach Discomfort	645	17.5%	652	17.7%	1500	18.2%	1492	18.1%	1398	18.4%	1404	18.5%
Peripheral Vascular Disease	1607	43.5%	1615	43.8%	4005	48.7%	3986	48.5%	3742	49.2%	3755	49.4%
Transient Ischemic Attack	255	6.9%	236	6.4%	647	7.9%	663	8.1%	596	7.8%	599	7.9%
Coronary Artery Disease	1347	36.5%	1361	36.9%	3331	40.5%	3311	40.3%	3126	41.1%	3146	41.4%
Baseline Medication Use												
Angiotensin Converting Enzyme Inhibitor	1285	34.8%	1264	34.2%	2938	35.7%	2950	35.9%	2714	35.7%	2696	35.4%
Amiodarone	340	9.2%	336	9.1%	814	9.9%	843	10.2%	765	10.1%	772	10.1%
Angiotensin Receptor Blocker	930	25.2%	958	26.0%	1993	24.2%	1990	24.2%	1921	25.3%	1983	26.1%
Beta Blockers	2509	68.0%	2540	68.8%	5659	68.8%	5702	69.3%	5304	69.7%	5286	69.5%
H2-receptor Antagonist	203	5.5%	228	6.2%	597	7.3%	597	7.3%	521	6.8%	521	6.8%
Proton Pump Inhibitor	1324	35.9%	1345	36.4%	2972	36.1%	2964	36.0%	2817	37.0%	2813	37.0%
Anti-platelets	836	22.6%	818	22.2%	1718	20.9%	1705	20.7%	1668	21.9%	1740	22.9%
Statins	2188	59.3%	2182	59.1%	4897	59.5%	4903	59.6%	4572	60.1%	4616	60.7%
Dronedarone	106	2.9%	112	3.0%	157	1.9%	182	2.2%	151	2.0%	140	1.8%
Calcium Channel Blockers	1432	38.8%	1457	39.5%	3189	38.8%	3156	38.4%	3001	39.5%	3000	39.4%
Baseline Hospitalization	1387	37.6%	1368	37.1%	3559	43.3%	3612	43.9%	3217	42.3%	3237	42.6%
Dosage on Index Date												
Standard			3125	84.7%			5665	68.9%			5714	75.1%
Follow-up Time (in days)												
minimum	1		1		1		1		1		1	
Q1	56		63		57		61		57		64	
median	148		163		150		177		153		161	
Q3	358		436		354		411		359		333	
maximum	1001		999		1001		1002		1001		951	

*SD* Standard deviation, *SE* Systemic embolism, *CHADS*_*2*_ Congestive heart failure, hypertension, age ≥ 75 years, diabetes mellitus, prior stroke or transient ischemic attack or thromboembolism; *CHA*_*2*_*DS*_*2*_*VAS*_*C*_ Congestive heart failure, hypertension, age ≥ 75 years, diabetes mellitus, prior stroke or transient ischemic attack or thromboembolism, vascular disease, age 65–74 years, gender category; *HAS-BLED* Hypertension, abnormal renal and liver function, stroke, bleeding, labile INRs (international normalized ratio), elderly, drugs and alcohol, *ACE* Angiotensin-converting enzyme inhibitor, *ARB* Angiotensin-receptor blocker, *NSAIDs* Non-steroidal anti-inflammatory drugs

### Effectiveness outcomes

**Fig. 2:**
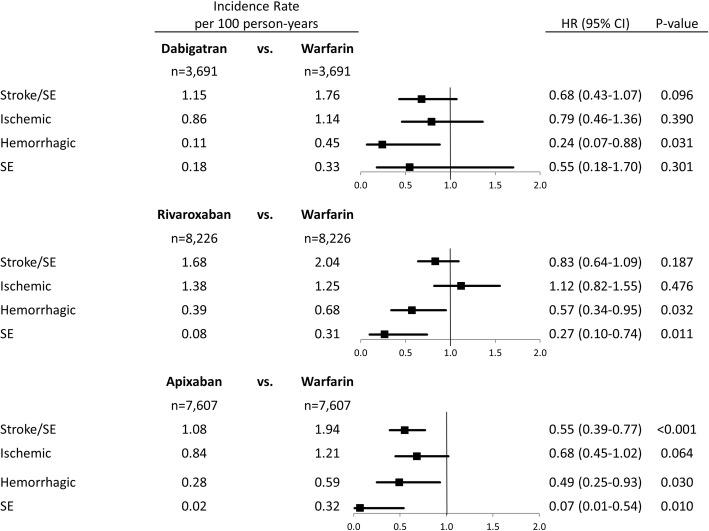
Propensity Score Matched Incidence Rates and Hazard Ratios for Stroke/SE. CI: confidence interval. SE: systemic embolism

The incidence rates of stroke/SE are shown in Fig. 2. Apixaban (hazard ratio [HR]: 0.55; 95% CI: 0.39, 0.77; *p* < 0.001) was associated with a significantly lower risk of stroke/SE compared to warfarin. Apixaban was also associated with a significantly lower risk of hemorrhagic stroke (HR: 0.49; 95% CI: 0.25, 0.93; *p* = 0.030) and SE (HR: 0.07; 95% CI: 0.01, 0.54; *p* = 0.010).

Compared to warfarin, dabigatran (HR: 0.68; 95% CI: 0.43, 1.07; *p* = 0.096) and rivaroxaban (HR: 0.83; 95% CI: 0.64, 1.09; *p* = 0.187) were associated with a non-significantly lower risk of stroke/SE (Fig. 2). Both dabigatran (HR: 0.24; 95% CI: 0.07, 0.88; *p* = 0.031) and rivaroxaban (HR: 0.57; 95% CI: 0.34, 0.95; *p* = 0.032) had a lower risk of hemorrhagic stroke versus warfarin.

### Safety outcomes

**Fig. 3:**
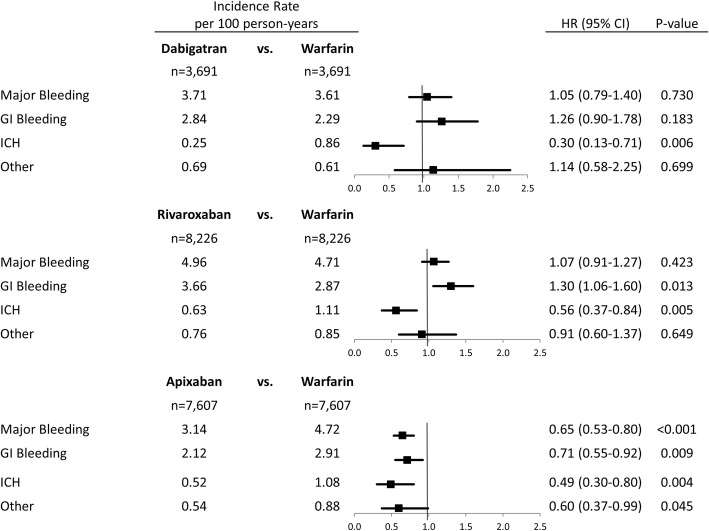
Propensity Score Matched Incidence Rates and Hazard Ratios for Major Bleeding. CI: confidence interval. GI: gastrointestinal. ICH: intracranial hemorrhage

The incidence rates of major bleeding are shown in Fig. 3. Apixaban (HR: 0.65; 95% CI: 0.53, 0.80; *p* < 0.001) patients had a significantly lower risk of major bleeding compared to warfarin. The decrease in major bleeding risk was driven by all types of major bleeding, including GI, ICH, and other major bleeding. Dabigatran (HR: 1.05; 95% CI: 0.79, 1.40; *p* = 0.730) and rivaroxaban (HR: 1.07; 95% CI: 0.91, 1.27; *p* = 0.423) were associated with similar risks of major bleeding compared to warfarin (Fig. 3). Both dabigatran (HR: 0.30; 95% CI: 0.13, 0.71; *p* = 0.006) and rivaroxaban (HR: 0.56; 95% CI: 0.37, 0.84; *p* = 0.005) were associated with a significantly lower risk of ICH versus warfarin; however, rivaroxaban was associated with a significantly higher risk of GI bleeding (HR: 1.30; 95% CI: 1.06,1.60; *p* = 0.013).

### Sensitivity analyses

The results were generally consistent in the dose subgroup analysis compared to the results in the main analysis (Table 2). There was a significant interaction for rivaroxaban treatment dose and major bleeding. The second and third sensitivity analyses showed results similar to the results of the main analysis. In the sensitivity analysis where patients with cardioversion and catheter ablation (i.e., low risk stroke patients) were excluded, patients who initiated rivaroxaban had a significantly lower risk of stroke/SE compared to patients who initiated warfarin (Table 3). All other trends remained the same.

**Table 2:** Dose Sensitivity Analysis for Propensity Score Matched Patients

	Dabigatran vs Warfarin		*P* value*	Rivaroxaban vs Warfarin		*P* value*	Apixaban vs Warfarin		*P* value*
Stroke/SE									
Reduced Dose	*N* = 566 vs *N* = 566	0.72 (0.25, 2.04)	0.857	*N* = 2561 vs *N* = 2561	0.77 (0.49, 1.20)	0.694	*N* = 1893 vs *N* = 1893	0.72 (0.38, 1.39)	0.315
Standard Dose	*N* = 3125 vs *N* = 3125	0.64 (0.39, 1.07)		*N* = 5665 vs *N* = 5665	0.86 (0.61, 1.22)		*N* = 5714 vs *N* = 5714	0.49 (0.32, 0.73)	
Major bleeding									
Reduced Dose	*N* = 566 vs *N* = 566	1.30 (0.70, 2.41)	0.369	*N* = 2561 vs *N* = 2561	0.84 (0.63, 1.12)	0.054	*N* = 1893 vs *N* = 1893	0.66 (0.46, 0.95)	0.803
Standard Dose	*N* = 3125 vs *N* = 3125	0.94 (0.68, 1.31)		*N* = 5665 vs *N* = 5665	1.19 (0.97, 1.47)		*N* = 5714 vs *N* = 5714	0.62 (0.48, 0.81)	

* *P*-value is for interaction. *CI* Confidence interval, *HR* Hazard ratio

**Table 3:** Other Sensitivity Analyses for Propensity Score Matched Patients

	Dabigatran vs Warfarin	Rivaroxaban vs Warfarin	Apixaban vs Warfarin
Censoring at 6 Months, HR (95% CI)			
Sample Size	*N* = 3691 vs N = 3691	*N* = 8226 vs *N* = 8226	*N* = 7607 vs *N* = 7607
Stroke/SE	0.68 (0.37–1.23)	0.90 (0.63–1.29)	0.51 (0.33–0.79)
Major bleeding	1.10 (0.75–1.61)	1.12 (0.91–1.39)	0.59 (0.45–0.77)
Intent-to-Treat, HR (95% CI)			
Sample Size	*N* = 3691 vs *N* = 3691	*N* = 8226 vs *N* = 8226	*N* = 7607 vs *N* = 7607
Stroke/SE	0.76 (0.57–1.03)	1.04 (0.86–1.26)	0.63 (0.49–0.81)
Major bleeding	0.97 (0.80–1.19)	1.01 (0.89–1.15)	0.75 (0.64–0.88)
Excluding Patients with Catheter Ablation or Cardioversion, HR (95% CI)			
Sample Size	*N* = 3298 vs *N* = 3298	*N* = 7698 vs *N* = 7698	*N* = 7034 vs *N* = 7034
Stroke/SE	0.68 (0.43–1.07)	0.83 (0.64–1.09)	0.55 (0.39–0.77)
Major bleeding	1.05 (0.79–1.40)	1.07 (0.91–1.27)	0.65 (0.53–0.80)

*CI* Confidence interval, *HR* Hazard ratio, *SE* Systemic embolism

## Discussion

In this real-world study among NVAF patients initiating OAC treatment in the US DOD population, apixaban was found to be the only DOAC associated with a significantly lower risk of stroke/SE and major bleeding compared to warfarin, while dabigatran and rivaroxaban initiation were associated with similar risk of stroke/SE and major bleeding compared to warfarin. These findings were supported by several sensitivity analyses.

This observational study adds real-world evidence to supplement the results from clinical trials. In the RE-LY trial, compared to warfarin, 110 mg dabigatran (not approved in the US) was associated with similar risk of stroke/SE and lower risk of major bleeding, while 150 mg dabigatran had a significantly lower risk of stroke/SE and similar risk of major bleeding [6]. However, in our study, we observed a similar risk of stroke/SE and major bleeding among dabigatran patients compared to warfarin patients. In the ROCKET-AF clinical trial, rivaroxaban was non-inferior for both stroke/SE and major bleeding compared to warfarin [7]. Similarly, our study showed a consistent safety result but numerically lower effectiveness results comparing rivaroxaban and warfarin. Consistent with our study, the ARISTOTLE trial found apixaban showed superiority to warfarin in terms of the risk of stroke/SE and major bleeding [8].

In addition to clinical trials, a few real-world studies have also examined the risk of stroke and major bleeding of OACs. In prior effectiveness and safety comparisons between dabigatran and warfarin, dabigatran was shown to have similar to lower risk of stroke/SE and major bleeding versus warfarin. In the Villines et al. study, which also used US DOD data, dabigatran was shown to be associated with a lower risk of stroke and similar risk of major bleeding compared to warfarin [25]. Consistent with the Villines et al. study, in a study using Medicare data, dabigatran was associated with a lower risk of ischemic stroke and similar risk of major bleeding compared to warfarin [11]. In a meta-analysis including 20 observational studies comparing dabigatran and warfarin, dabigatran was found to have a lower risk of ischemic stroke and major bleeding [26]. However, another meta-analysis found no statistical difference between dabigatran and VKA for ischemic stroke or major bleeding [10] Dabigatran 110 mg is not available in the US; therefore, this study included patients prescribed 150 mg or 75 mg dabigatran and may not be generalizable to countries where 110 mg dabigatran is available. Our study indicated that dabigatran had a numerically lower risk of stroke/SE and similar risk of major bleeding compared to warfarin. Since the dabigatran cohort has the smallest sample size in our study, a larger sample size may be warranted for this population to examine the difference between dabigatran and warfarin.

In many real-world comparisons of rivaroxaban and warfarin, rivaroxaban was associated with a similar risk of stroke/SE and major bleeding compared to warfarin; however, some inconsistencies exist in other real-world studies. In a meta-analysis of observational study, Ntaios et al. found that there was no statistical difference between rivaroxaban and VKA for stroke/SE and major hemorrhage [10]. However, in a meta-analysis, Bai et al. found that rivaroxaban was associated with a lower risk of stroke/SE and a similar risk of major bleeding [27]. In Amin et al. (Medicare data), rivaroxaban was associated with a lower risk of stroke/SE, but a higher risk of major bleeding compared to warfarin [17]. Tamayo et al. evaluated major bleeding incidence rates among rivaroxaban users in the DOD population and found the incidence of major bleeding to be 2.86 per 100 person-years. The reported incidence is smaller than our study where the incidence of major bleeding was 4.96 per 100 person-years. The difference may have been due to different selection criteria; for example, we excluded patients with previous anticoagulant use, and we used a different definition of major bleeding [28].

In this study, apixaban was the only DOAC that showed significant safety and effectiveness results, which is generally consistent with other real-world studies. Similarly, in a study pooling four claims datasets, apixaban initiators were associated with a 33% lower risk of stroke/SE and 40% lower risk of major bleeding compared with warfarin initiators [29]. In the Amin et al. study using Medicare data, apixaban was also associated with both significantly lower risk of stroke/SE and major bleeding compared to warfarin [17]. The Ntaios et al. meta-analysis demonstrated that apixaban was associated with a similar risk of ischemic stroke/SE and lower risk of major hemorrhage compared to warfarin [10]. Another meta-analysis of apixaban and warfarin comparisons showed that apixaban had similar risk of stroke/SE and lower risk of major bleeding versus warfarin [30].

By comparing the effectiveness and safety of DOACs versus warfarin using the most recent DOD data, this study provides supplemental information for the clinical trials as well as the real-world study profiles. To our knowledge, this was the first study using DOD data to examine the effectiveness and safety of all DOACs compared to warfarin. Findings from this study may inform decision makers and health care providers in the DOD and other health care systems.

This study has several limitations. First, due to the nature of claims studies, diagnoses and procedures in this study were identified using ICD-9-CM, CPT, HCPCS, and NDC codes. These coding systems were originally designed for billing purposes rather than research, without further adjudication using precise clinical criteria. Second, although cohorts were PSM, potential residual confounders exist hence no causal inferences can be drawn. In addition, since PSM was conducted between each DOAC and warfarin, no comparisons across the three DOACs should be made. Although no direct comparison to the clinical trials can be made given the nature of observational study, our findings from the main, subgroup, and sensitivity analyses provided additional real-world evidence and support for the clinical trial study results. Finally, only treatment-naïve patients and the DOD population were evaluated in the study, which may impact the generalization of the results.

## Conclusions

This analysis using the US DOD data adds real-world evidence about the comparative effectiveness and safety of OAC use for stroke prevention in NVAF. Among NVAF patients in the US DOD population, apixaban initiation was associated with significantly lower risks of stroke/SE and major bleeding compared to warfarin. Dabigatran and rivaroxaban initiation were associated with similar risks of stroke/SE and major bleeding compared to warfarin.

## Additional file

Additional file 1: **Table S1.** Codes for Exclusion Criteria. **Table S2.** ICD-9-CM Codes for Stroke/SE and Major Bleeding Endpoints. **Table S3.** CHA_2_DS_2_-VASc Score Points and Description. **Table 4.** HAS-BLED Score. (DOCX 26 kb)

## Data Availability

The datasets generated during and/or analyzed during the current study are not publicly available due to a data licensing agreement with the United States Department of Defense. The raw data on which the analysis was based are available from the US Department of Defense on reasonable request.

## Abbreviations

AF: Atrial fibrillation
ARB: Angiotensin-receptor blocker
CCI: Charlson comorbidity index
CHA_2_DS_2_VASC: Congestive heart failure, hypertension, age ≥ 75 years, diabetes mellitus, prior stroke or transient ischemic attack or thromboembolism, vascular disease, age 65–74 years, gender category
CHADS_2_: Congestive heart failure, hypertension, age ≥ 75 years, diabetes mellitus, prior stroke or transient ischemic attack or thromboembolism
CI: Confidence interval
CPT: Current procedural terminology
DOAC: Direct Oral anticoagulant
DOD: Department of Defense
FDA: Food and Drug Administration
GI: Gastrointestinal
HAS-BLED: Hypertension, abnormal renal and liver function, stroke, bleeding, labile INRs (international normalized ratio), elderly, drugs and alcohol
ACE: Angiotensin-converting enzyme inhibitor
HCPCS: Healthcare common procedure coding system
HR: Hazard ratio
ICD-9-CM: International classification of diseases, 9th revision, clinical modification
ICH: Intracranial hemorrhage
INR: International normalized ratio
NDC: National Drug Code
NVAF: Non-valvular atrial fibrillation
OAC: Oral anticoagulant
PSM: Propensity score matching
SD: Standard deviation
SE: Systemic embolism
TIA: Transient ischemic attack
VKA: Vitamin K antagonist
