# Supplementary material for: Effectiveness and safety of direct oral anticoagulants compared to warfarin in treatment naïve non-valvular atrial fibrillation patients in the US Department of defense population
Source: BMC Cardiovasc Disord. 2019 Jun 13;19:142. doi: 10.1186/s12872-019-1116-1 (PMC6567643; doi:10.1186/s12872-019-1116-1)
Supplement: Supplementary file 1 — Table S1. Codes for Exclusion Criteria. Table S2. ICD-9-CM Codes for Stroke/SE and Major Bleeding Endpoints. Table S3. CHA2DS2-VASc Score Points and Description. Table 4. HAS-BLED Score. (DOCX 26 kb) [file 12872_2019_1116_MOESM1_ESM.docx]

**Supplemental Table 1. Codes for Exclusion Criteria**

| **Diagnosis** | **ICD-9 Codes or HCPCS Codes** |
| --- | --- |
| Rheumatic mitral valvular heart disease, mitral valve stenosis | 394.0, 394.1, 394.2, 394.9, 396.0, 396.1, 396.8, 396.9, 424.0, 745.xx |
| Heart valve replacement or surgery | V422, V433, 35.05-35.09, 35.20-35.28 and 35.97 |
| Dialysis, kidney transplant, end-stage chronic kidney disease | 585.6, 996.73, V45.1x, V56.x; 55.6x; CPT: 90935, 90937, 90945, 90947, 90967; 00868, 50300, 50320, 50323, 50325, 50327, 50328, 50329, 50340, 50360, 50365, 50380 |
| VTE | 451-453, 671.3, 671.4, 671.9, 415.1, 673.2, 673.8 |
| Reversible AF (Heart valve replacement/transplant, pericarditis, thyrotoxicity | Pericarditis: 006.8, 017.9, 036.41, 074.21, 093.81, 098.83, 115.93, 390, 391, 392.0, 393, 411.0, 420.90, 420.91, 420.99, 423.0, 423.1, 423.2, 423.8, 423.9  Thyrotoxicity: 242.0, 242.1, 242.2, 242.3, 242.4, 242.8, 242.9 |
| Hip or knee replacement | V43.64, V43.65, 81.40, 81.51, 81.52, 81.53, 81.54, 81.55 |
| Pregnancy | ICD-9-CM codes 630-679, V22, V23, V24, V27, V28, V61.6, V61.7, 792.3, 796.5, 72-75.99  HCPCS codes 59000-59350, 76801-76828, 83661-83664 |

**Supplemental Table 2. ICD-9-CM Codes for Stroke/SE and Major Bleeding Endpoints**

| **Stroke** | **ICD-9-CM** | **Description** |
| --- | --- | --- |
| **Hemorrhagic Stroke** | 430 | Subarachnoid hemorrhage |
|  | 431 | Intracerebral hemorrhage |
|  | 432.x | Other and unspecified intracranial hemorrhage |
|  | Exclusion | Exclude above codes if traumatic brain injury (ICD-9-CM: 800-804, 850-854)is present during the hospitalization |
| **Ischemic Stroke** | 433.x1 | Occlusion and stenosis of precerebral arteries with cerebral infarction |
|  | 434.x1 | Occlusion of cerebral arteries with cerebral infarction |
|  | 436 | Acute, but ill-defined, cerebrovascular disease |
| **Systemic Embolism** | 444.x | Arterial embolism and thrombosis |
|  | 445.x | Atheroembolism |
| **Type of Major Bleeding** | **ICD-9 Code** | **Description** |
| **Major GI Bleeding** | 456.0x | Esophageal varices with bleeding |
|  | 456.20 | Esophageal varices in diseases classified elsewhere, with bleeding |
|  | 530.82 | Esophageal hemorrhage |
|  | 531.0x | Acute gastric ulcer with hemorrhage |
|  | 531.2x | Acute gastric ulcer with hemorrhage and perforation |
|  | 531.4x | Chronic or unspecified gastric ulcer with hemorrhage |
|  | 531.6x | Chronic or unspecified gastric ulcer with hemorrhage and perforation |
|  | 532.0x | Acute duodenal ulcer with hemorrhage |
|  | 532.2x | Acute duodenal ulcer with hemorrhage and perforation |
|  | 532.4x | Chronic or unspecified duodenal ulcer with hemorrhage |
|  | 532.6x | Chronic or unspecified duodenal ulcer with hemorrhage and perforation |
|  | 533.0x | Acute peptic ulcer of unspecified site with hemorrhage |
|  | 533.2x | Acute peptic ulcer of unspecified site with hemorrhage and perforation |
|  | 533.4x | Chronic or unspecified peptic ulcer of unspecified site with hemorrhage |
|  | 533.6x | Chronic or unspecified peptic ulcer of unspecified site with hemorrhage and perforation |
|  | 534.0x | Acute gastrojejunal ulcer with hemorrhage |
|  | 534.2x | Acute gastrojejunal ulcer with hemorrhage and perforation |
|  | 534.4x | Chronic or unspecified gastrojejunal ulcer with hemorrhage |
|  | 534.6x | Chronic or unspecified gastrojejunal ulcer with hemorrhage and perforation |
|  | 535.01 | Acute gastritis, with hemorrhage |
|  | 535.11 | Atrophic gastritis, with hemorrhage |
|  | 535.21 | Gastric mucosal hypertrophy, with hemorrhage |
|  | 535.31 | Alcoholic gastritis, with hemorrhage |
|  | 535.41 | Other specified gastritis, with hemorrhage |
|  | 535.51 | Unspecified gastritis and gastroduodenitis, with hemorrhage |
|  | 535.61 | Duodenitis, with hemorrhage |
|  | 537.83 | Angiodysplasia of stomach and duodenum with hemorrhage |
|  | 562.02 | Diverticulosis of small intestine with hemorrhage |
|  | 562.03 | Diverticulitis of small intestine with hemorrhage |
|  | 562.12 | Diverticulosis of colon with hemorrhage |
|  | 562.13 | Diverticulitis of colon with hemorrhage |
|  | 568.81 | Hemoperitoneum (nontraumatic) |
|  | 569.3 | Hemorrhage of rectum and anus |
|  | 569.85 | Angiodysplasia of intestine with hemorrhage |
|  | 578.x | Gastrointestinal hemorrhage |
|  | Procedure Codes |  |
|  | 44.43 | Endoscopic Control Of Gastric Or Duodenal Bleeding |
| **Intracranial Bleeding** | 430 | Subarachnoid hemorrhage |
|  | 431 | Intracerebral hemorrhage |
|  | 432.x | Other and unspecified intracranial hemorrhage |
|  | 852.0x | Subarachnoid hemorrhage following injury without mention of open intracranial wound |
|  | 852.2x | Subdural hemorrhage following injury without mention of open intracranial wound |
|  | 852.4x | Extradural hemorrhage following injury without mention of open intracranial wound |
|  | 853.0x | Other and unspecified intracranial hemorrhage following injury without mention of open intracranial wound |
| **Other Major Bleeding** | 285.1 | Decrease on Hb or Hematocrit |
|  | 360.43 | Hemophthalmos, except current injury |
|  | 362.43 | Hemorrhagic detachment of retinal pigment epithelium |
|  | 362.81 | Retinal hemorrhage |
|  | 363.61 | Choroidal hemorrhage, unspecified |
|  | 363.62 | Expulsive choroidal hemorrhage |
|  | 363.72 | Hemorrhagic choroidal detachment |
|  | 364.41 | Hyphema of iris and ciliary body |
|  | 372.72 | Conjunctival hemorrhage |
|  | 374.81 | Hemorrhage of eyelid |
|  | 376.32 | Orbital hemorrhage |
|  | 377.42 | Hemorrhage in optic nerve sheaths |
|  | 379.23 | Vitreous hemorrhage |
|  | 423.0x | Hemopericardium |
|  | 596.7x | Hemorrhage into bladder wall |
|  | 599.7x | Hematuria |
|  | 602.1x | Congestion or hemorrhage of prostate |
|  | 620.1 | Corpus luteum cyst or hematoma |
|  | 621.4 | Hematometra |
|  | 626.2 | Excessive or frequent menstruation |
|  | 626.5 | Ovulation bleeding |
|  | 626.7 | Postcoital bleeding |
|  | 626.8 | Other disorders of menstruation and other abnormal bleeding from female genital tract |
|  | 626.9 | Unspecified disorders of menstruation and other abnormal bleeding from female genital tract |
|  | 719.1x | Hemarthrosis |
|  | 782.7 | Spontaneous ecchymoses |
|  | 784.7 | Epistaxis |
|  | 784.8 | Hemorrhage from throat |
|  | 786.3x | Hemoptysis |
|  | 958.2 | Secondary and recurrent hemorrhage |
|  | 997.02 | Iatrogenic cerebrovascular infarction or hemorrhage |
|  | 998.11 | Hemorrhage complicating a procedure |
|  | Procedure Codes |  |
|  | 99.04 | Transfusion Of Packed Cells |

**Supplemental Table 3. CHA_2_DS_2_-VASc Score Points and Description**

| Condition | ICD-9-CM Codes / Criteria | Point |
| --- | --- | --- |
| **C**ongestive heart failure | 398.91, 402.x1, 404.x3, 428.xx | 1 |
| **H**ypertension | 401.xx-405.xx | 1 |
| **A**ge | ≥75 years | 2 |
| **D**iabetes | 250.xx, 357.2, 362.0, 366.41 | 1 |
| **S**troke (non-hemorrhagic only & transient ischemic attack) | V12.54, 433.xx-435.xx | 2 |
| **V**ascular disease (myocardial infarction, peripheral arterial disease, aortic plaque) | 410.xx, 412, 440.xx, 441.xx, 442.xx, 443.xx, 444.2x, 445.0x | 1 |
| **A**ge | 65-74 years | 1 |
| **S**ex **C**ategory | Female | 1 |

**Supplemental Table 4. HAS-BLED Score**

| **HAS-BLED score** | **Codes** | **Points Algorithm** |
| --- | --- | --- |
| Hypertension | Hypertension 401.xx-405.xx | 1 point |
| Abnormal kidney and/or liver function: | Kidney: 580.xx-589.xx Liver: 570.xx-573.xx | 1 point each |
| Stroke | History of stroke V12.54, 433.xx-435.xx | 1 point |
| Bleeding | Baseline bleeding (Table 11), Anemia 280.xx-286.xx | 1 point |
| Labile INR | Not measurable. | Not applicable |
| Elderly | 65+ years | 1 point for age 65 or older |
| Alcohol/ Drug Therapy | 303.xx, 305.0x, V11.3x | 1 point |
|  | Antiplatelets administered (abciximab, anagrelide HCL, aspirin, aspirin/dipyridamole, cilostazol, clopidogrel, dipyridamole, eptifibatide, prasugrel, ticagrelor, ticlopidine, tirofiban) NSAIDs administered (bromfenac, celecoxib, diclofenac, etodolac, fenoprofen, flurbiprofen, ibuprofen, indomethacin, ketoprofen, ketorolac, lansoprazole/naproxen, meclofenamate, mefenamic acid, meloxicam, nabumetone, naproxen, oxaprozin, piroxicam, sulindac, tolmetin) | 1 point |
